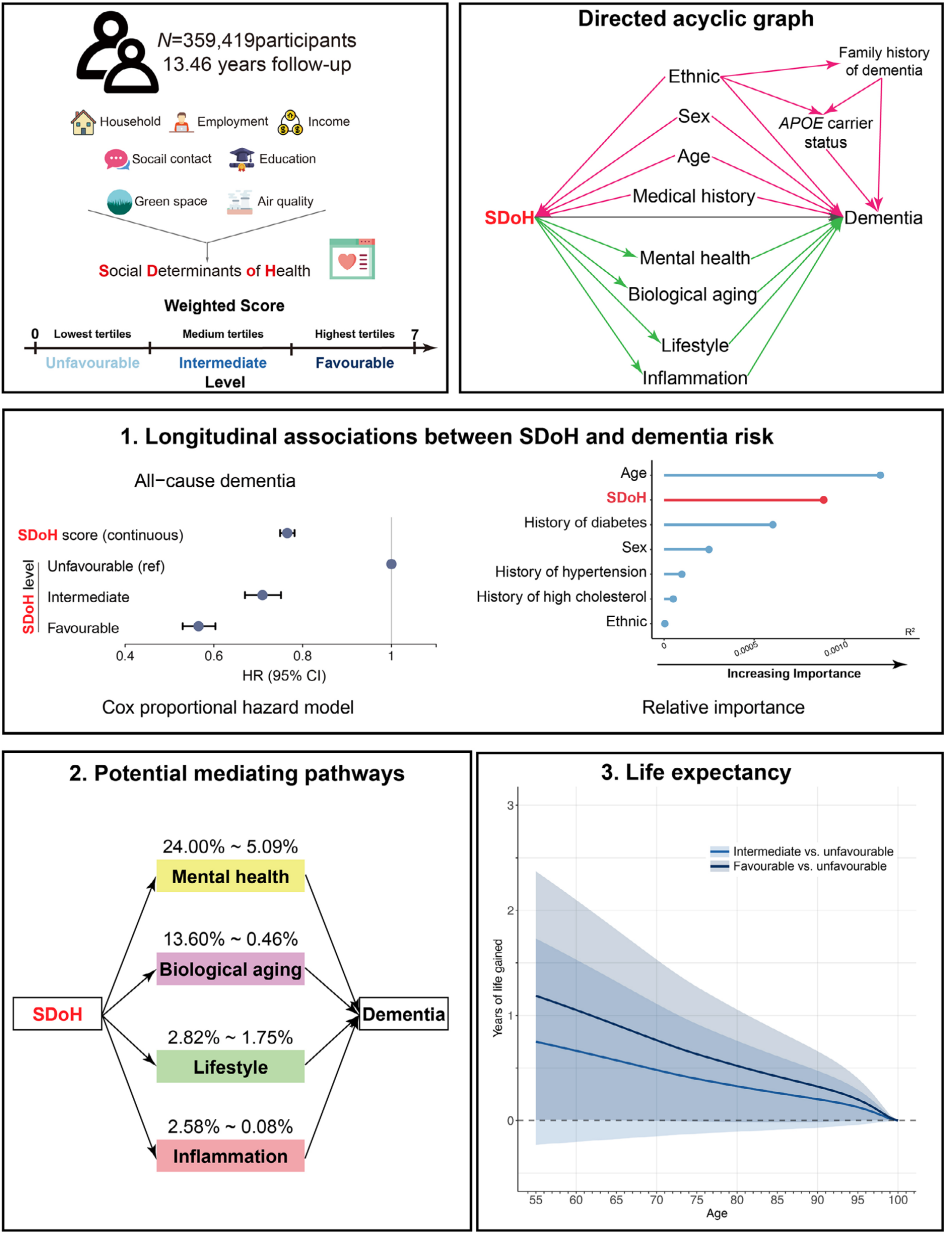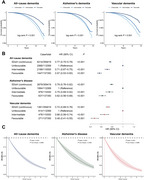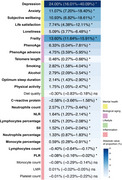# Mental health, biological aging, and lifestyle mediate the associations between social determinants of health and dementia

**DOI:** 10.1002/alz70862_110855

**Published:** 2025-12-23

**Authors:** Pei‐Yang Gao, Jie Chang, Yi Tang

**Affiliations:** ^1^ Xuanwu Hospital Capital Medical University, Beijing, Beijing China; ^2^ Department of Neurology & Innovation Center for Neurological Disorders, Xuanwu Hospital, Capital Medical University, National Center for Neurological Disorders, Beijing, Beijing China

## Abstract

**Background:**

Social determinants of health (SDoH) represent multiple interconnected social and economic factors that have been proposed by the World Health Organization as critical non‐medical influences on health outcomes. However, few studies have investigated the relationships and potential mechanisms between SDoH and dementia in large‐scale population‐based cohorts.

**Method:**

This population‐based prospective study utilized data from the UK Biobank. Our study incorporated seven factors acrossfour domains—economic stability, educational attainment, social contact, and environmental factors—to assess SDoH levels. Cox proportional hazard models and linear regression models were conducted to examine the relationship between SDoH and risk of all‐cause and cause‐specific dementia, brain structure (focusing on hippocampal volume), and cognitive performance. Causal mediation analysis explored potential mediating factors, including mental health, biological aging, and lifestyle, linking SDoH and dementia. Survival models were applied to estimate differences in life expectancy.

**Result:**

Among 359,419 participants (median follow‐up: 13.46 years), those with favorable SDoH levels demonstrated significantly lower risks of developing all‐cause dementia (HR: 0.57, 95% CI: 0.53–0.60), Alzheimer's disease (HR: 0.62, 95% CI: 0.56–0.68), and vascular dementia (HR: 0.48, 95% CI: 0.41–0.55) compared to those with unfavorable SDoH levels. In terms of impact on dementia risk, SDoH ranked second only to age among all analyzed factors. Higher SDoH levels were positively associated with larger hippocampal volume and better performance across cognitive domains. The relationship between SDoH and dementia was mediated by multiple factors: depression (24.00%), frailty (13.60%), biological aging measured by PhenoAge (6.33%), and smoking (2.82%). At age 65, participants with dementia who had favorable SDoH levels demonstrated an average life expectancy 0.909 (95% CI 0.002–1.873) years longer than those with unfavorable SDoH levels.

**Conclusion:**

Higher levels of SDoH are associated with a reduced risk of dementia, and the associations are mediated by mental health, biological aging, and lifestyle. These findings highlight that prioritizing improvements in SDoH should be a key component of global efforts aimed at mitigating dementia risk and promoting cognition and longer lifespans across populations.